# Era of molecular diagnosis for pathogen identification of unexplained pneumonia, lessons to be learned

**DOI:** 10.1080/22221751.2020.1738905

**Published:** 2020-03-16

**Authors:** Jing-Wen Ai, Yi Zhang, Hao-Cheng Zhang, Teng Xu, Wen-Hong Zhang

**Affiliations:** a Department of Infectious Diseases, National Clinical Research Center for Aging and Medicine, Huashan Hospital, State Key Laboratory of Genetic Engineering, School of Life Science, Key Laboratory of Medical Molecular Virology (MOE/MOH) and Institutes of Biomedical Sciences, Shanghai Medical College, Fudan University, Shanghai, People’s Republic of China; b Vision Medicals Co., Ltd, Guangzhou, People’s Republic of China

**Keywords:** Unexplained pneumonia, diagnosis, SARS-CoV-2, COVID-19, molecular

## Abstract

Unexplained pneumonia (UP) caused by a novel coronavirus SARS-CoV-2 (severe acute respiratory syndrome coronavirus 2) emerged in China in late December 2019 and has infected more than 9000 cases by 31 January 2020. Shanghai reported the first imported case of COVID-19 (Coronavirus Disease 2019) in 20 January 2020. A combinative approach of real-time RT–PCR, CRISPR-based assay and metagenomic next-generation sequencing (mNGS) were used to diagnose this unexplained pneumonia patient. Real-time RT–PCR and CRISPR-based assay both reported positive. This sample belonged to Betacoronavirus and shared a more than 99% nucleotide (nt) identity with the Wuhan SARS-CoV-2 isolates. We further compared pros and cons of common molecular diagnostics in UP. In this study, we illustrated the importance of combining molecular diagnostics to rule out common pathogens and performed mNGS to obtain unbiased potential pathogen result for the diagnosis of UP.

Unexplained pneumonia (UP) was defined as pneumonia without confirmed diagnosis. In late December 2019, Chinese social media first released news concerning the possible severe acute respiratory syndrome (SARS) virus infected cases in Wuhan. On 31 December 2019, the Health Commission of Hubei province, China announced a cluster of unexplained pneumonia (UP) and later a novel coronavirus SARS-CoV-2 as named by the WHO was declared to be the causative agent[1,2]. During the following month, multiple SARS-CoV-2 cases were gradually reported all over China and around 9000 definite cases were declared by 31 January 2020 [3]. As of today, case reports are still escalating on a daily basis. The reasons behind this outbreak are numerous, the highly infectious nature of SARS-CoV-2, limited pathogen detection method for unexplained pneumonia, the high population density of the Hubei Province and across China, etc. Various diagnostic methods were used during the initial search of this novel virus, yet their pros and cons have not been discussed thoroughly.

On 20 January, Shanghai reported the first imported case of SARS-CoV-2 infection, and through this case, we seek a combinative approach of selected techniques to improve pathogen identification of unexplained pneumonia in the future.

The patient was a 56 years old woman and came to Shanghai from Wuhan on 12 January. She went to the fever clinic of a local hospital due to fever and fatigue. The patient had relatively lower lymphocyte count. Under the close collaboration with the Shanghai CDC, we first performed FilmArray multiplex PCR respiratory panel 2.0 (Biomerieux, Utah, USA) with negative results. We then performed real-time RT–PCR, CRISPR-based assay and metagenomic next-generation sequencing (mNGS) on her respiratory sample and finally diagnosed her with COVID-19.

Total RNA was extracted from the patient’s pharyngeal swab using QIAamp Viral RNA Mini Kit (QIAGEN, Germany). We performed Real-time PCR for this patient as described [4]. CRISPR-nCoV was performed using CRISPR-nCoV kit from Vision Medicals, China. The RNA sample was also prepared for high-throughput mNGS. The qualified library was sequenced on an Illumina Nextseq using a single-end mode (1 × 75bp). Low-quality reads and reads derived from human genome sequences were removed. After that, de novo assembly was done using SPAdes. 29 genomes of coronaviruses and 2 SARS-CoV-2 genomes released by China were downloaded from NCBI for phylogenetic analysis. Phylogenetic tree was built according to the N gene using multiple sequence alignment algorithm ClustalX2 [5]. Phylogenetic tree was then built through MEGA X[6]. Meanwhile, we downloaded a total of 13 β family coronavirus genomes from NCBI and used pyani (https://pypi.org/project/pyani/) for homology analysis.

A positive result of SARS-CoV-2 was reported by RT–PCR. As illustrated from Figure 1(A), Ct value was 24.69 while that of the positive control was 25.97. CRISPR-nCoV was also positive with a fold-change value of 24 compared to the negative control. As shown in Figure 1(B), the sequence homology among the sample and other coronaviruses were assessed. Classified as subgenus *Sarbecovirus* of the genus Betacoronavirus, this sample shared a great nucleotide (nt) identity (>99%) with the two SARS-CoV-2 isolates and was 100% identical with that of SARS-CoV-2 BetaCoV/Wuhan/IPBCAMS-WH-01/2019 (GenBank MT019529.1). By contrast, a substantially lower nt identity of 84% was found with SARS-CoV (GenBank NC004718.3). The genome mapping coverage with SARS-CoV-2 Wuhan-Hu-1 (GenBank MN908947.3) was 85.98% and 33.35x in depth (Figure 1(C)). In analysis of complete genome similarity in Figure 1(D), we revealed a high similarity between this sample and two SARS-CoV-2 isolates.

**Figure 1.**
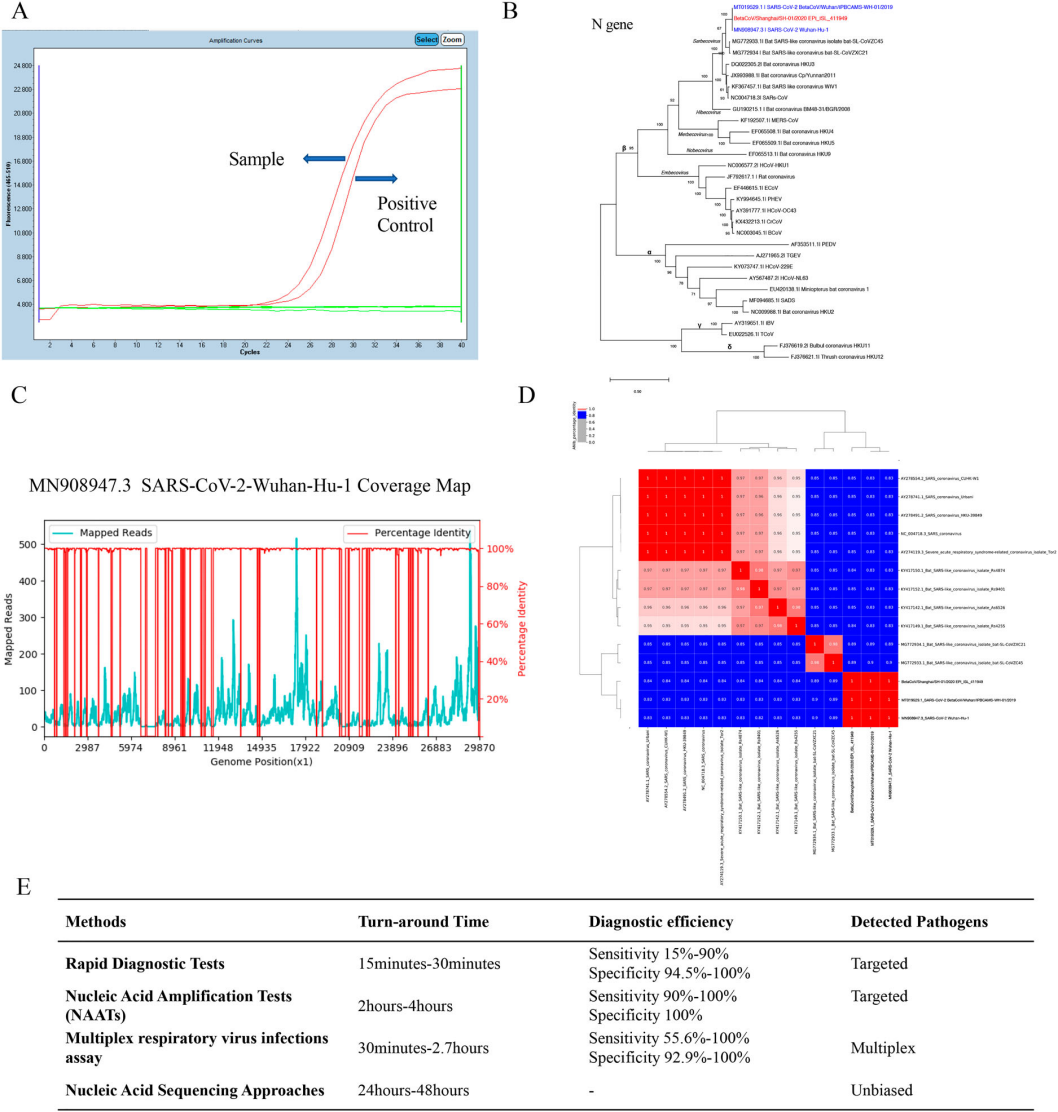
Molecular diagnostic approaches in this patient and comparisons of updated diagnostic methods. (A) Real-time amplification curve for detecting SARS-CoV-2. The left curve pointed to this sample and the right one meant the positive control. (B) Phylogenetic tree based on N gene of coronavirus including SARS-CoV-2 genomes (blue labels) and this sample (red label). (C) Genome coverage and depth compared with SARS-CoV-2 Wuhan-Hu-1 (GenBank MN908947.2) genome. (D) Genome similarity analysis with other 13 β family coronaviruses. The numbers showed the percentage of nt identity. (E) Comparisons of characteristics of updated diagnostic methods including rapid diagnostic tests (RDT), NAATs, multiplex respiratory virus infection assays and mNGS.

Ever since the diagnosis of pathogen reached the era of molecular approaches, a variety of methods have showed to increase diagnostic efficacy. In this particular case, RT–PCR, CRISPR, and metagenomics sequencing have all exhibited their potential in the rapid diagnosis of newly emerging microorganism. However, lessons should be learned from this outbreak.

Acute viral respiratory tract infection is one of the major causes of unexplained pneumonia, especially in winter. Culture, as the widely recognized gold standard, is time-consuming and labour intensive. Conventional diagnostic methods such as direct/indirect immunofluorescence assay (IFA) and rapid antigen detection also have their own significant limitations.

What’s more, various culture-independent nucleic acid amplification tests (NAATs) and point-of-care tests (POCT) could aid the diagnosis of unexplained pneumonia, including polymerase chain reaction (PCR), loop-mediated isothermal amplification (LAMP), clustered regularly interspaced short palindromic repeats (CRISPR), etc. [7]. However, these methods were not able to detect novel pathogens as in the case of Hubei Province. The latest multiplex respiratory virus infections assay includes Xpert Xpress Flu/RSV assay (GeneXpert System, Cepheid, Sunnyvale, CA, USA), Qiagen ResPlex II V2.0 kit and FilmArray multiplex PCR system [7]. These methods offered better diagnostic rates for UP by broadening pathogen coverage. Despite the lack of capability when it comes to identifying novel pathogens. these syndromic tests are still valuable for ruling out the common pathogens in the first-line diagnosis of unexplained pneumonia, as reported in the articles revolving around the SARS-CoV-2 outbreaks.

At present, several companies have developed and produced virus assays (Supplementary material) for SARS-CoV-2 and its typical Betacoronavirus organization consists of a 5′ untranslated region (UTR), replicase complex (orf1ab), S gene, E gene, M gene, N gene, 3′ UTR, and several unidentified nonstructural open reading frames [8]. The current nucleic acid amplification tests methods mainly targeted the open reading frames of the replicase complex (orf1ab), S, E, M and N genes. Besides various NAATs, a new rapid antigen detection assay has also been developed. In order to put diagnostic assays into use as soon as possible, Hubei Province has adopted a trial-and-request approach, which allows trials at medical institutions and review procedures after epidemic situation is resolved.

Shotgun metagenomics sequencing including short-read and long-read sequencing could obtain genomic data from both known and novel pathogens. mNGS has been used in investigations of COVID-19 outbreaks after exclusion of common pathogens by NAATs [9–11]. Current bioinformatic pipeline for metagenomics works by mapping the sequenced reads to an existing database of previously known microbes. This approach may result in partial alignment of a novel pathogen genome to that of some previously identified microbes and lead to incorrect taxonomic classification [12]. In fact, during the SARS-CoV-2 outbreak, one of the first news releases came from a report of commercial sequencing company stating their identification of SARS virus, causing wide panic among society. And only later after careful analysis on more sequencing data, the causative agent was found to be a novel species. The advantage of metagenomic sequencing lies in its unbiased nature which enables it to play a crucial role in the future diagnosis of the unexplained pneumonia, especially when encountering untypical causative pathogen. The alert should be raised that results from metagenomic sequencing should always be interpreted with the consultant of medical professionals, especially when dealing with a potentially novel pathogen or clustered cases.

In the present case, the combination of RT–PCR, CRISPR and mNGS confirmed our clinical diagnosis for COVID-19. This cross-validation was crucial at the early stage of the outbreak when our understandings on the novel pathogen were limited. For instance, binding of certain primers might be abrupted by genetic variants in the viral genome, resulting in false negative results. Moreover, by harnessing an unbiased metagenomic assay, the clinicians were also granted with information on potential concomitant infections to guide more appropriate treatment. Looking forward, as the CRISPR-based techniques further mature, we envisage its broader clinical uses for targeted, rapid pathogen detection especially in under-resourced settings where complicated PCR instruments may not be available. Despite its relatively longer turn-around time, metagenomics holds great promises as a unique diagnostic, especially for patients with acute or severe infections.

All these three methods consist of an identical RNA extraction procedure, which takes about an hour. The residual workflow of RT–PCR and CRISPR is library preparation and amplification, while the residual workflow of mNGS breaking into several part: library preparation (3 h), mNGS sequencing (18 h) and data analysis (3 h). Thus, turn-around time (TAT) of RT–PCR, CRISPR and mNGS is about 3, 2 and 24 h, respectively. RNA extraction is a necessity, the experimenters need to expose to the samples directly in the biohazard safety equipment with tertiary protection. We suppose there is no obvious difference in safety among these approaches. We further compared TAT, diagnostic efficiency and detected pathogens of updated diagnostic methods in UP in Figure 1(E) [7,13]. In the future, pros and cons of each molecular diagnostics should be thoroughly taken into consideration by the physicians to reach a more informational and accurate clinical diagnosis.

Here we reported the first definite case of COVID-19 in Shanghai, in which the virus was detected by employing various methods. This case illustrated the importance of combining targeted NAATs to rule out common pathogens and metagenomic assay to obtain unbiased genomic information on the pathogen for diagnosis of UP.

## Supplementary Material

Supplemental Material

## Data Availability

We have uploaded the assembled sequence to GISAID as EPI_ISL_411949.
